# Vascular Changes in the Macula and Peripapillary Regions Using Optical Coherence Tomography Angiography in Migraine with Aura and Migraine Without Aura

**DOI:** 10.3390/jcm15103853

**Published:** 2026-05-16

**Authors:** Patrycja Lesiuk, Anna K. Szewczyk, Krystyna Mitosek-Szewczyk, Agnieszka Brzozowska, Robert Rejdak, Katarzyna Nowomiejska

**Affiliations:** 1 Chair and Department of General and Pediatric Ophthalmology, Medical University of Lublin, 20-059 Lublin, Poland; 2 Department of Neurology, Medical University of Lublin, 20-059 Lublin, Poland; 3 Department of Neurology, University Clinical Hospital No. 4, 20-090 Lublin, Poland; 4 Department of Mathematics and Medical Biostatistics, Medical University of Lublin, 20-059 Lublin, Poland

**Keywords:** migraine, macula, optic coherence tomography angiography, vascular density

## Abstract

**Background:** The aim of this study was to assess the macular vasculature and retinal thickness using optical coherence tomography angiography (OCTA) in patients with migraine with aura (MA) and migraine without aura (MO) compared with healthy controls (HCs). **Methods:** A prospective cohort study included 37 MO patients, 34 MA patients, and 34 HCs. Vessel density (VD) in the superficial and deep retinal plexuses, radial peripapillary capillaries (RPC), optic nerve head (ONH), and foveal avascular zone (FAZ) was measured. The retinal thickness, retinal nerve fiber layer (RNFL) thickness, visual field parameters, and body mass index (BMI) were also evaluated. **Results:** Significantly lower macular superficial VD in the whole image was observed in the MA group compared with both the MO and HCs. MO patients showed higher RPC VD in the inferior quadrant compared with HCs. No significant differences were found in the RNFL thickness, deep plexus, FAZ area, non-flow area, or visual field parameters. In the MA group, the FAZ area showed a significant inverse correlation with the BMI, and a higher incidence of comorbidities was observed, particularly of Hashimoto’s thyroiditis. **Conclusions:** These findings suggest that migraine subtypes are associated with distinct retinal microvascular patterns detectable via OCTA. Reduced macular perfusion in MA and increased peripapillary perfusion in MO may reflect subtype-specific vascular dysregulation. OCTA may therefore serve as a noninvasive biomarker for detecting early microvascular alterations in migraine.

## 1. Introduction

Migraine is the most frequent neurological problem in primary care [1]. According to the findings of the last Global Burden Disease 2021 study, migraine continues to be second among the world’s causes of disability in young adults (15–49 years old) and the second cause of disability in the same group of age in Poland [2]. Migraine is a common condition, affecting 18% of women and 6% of men. The prevalence of migraine worldwide is estimated to be 12% of the global population [3] The peak age range for migraine is typically between 18 to 44 years. It is an extremely burdensome condition for the patients, their families, and the society [4]. Migraine is a severe neurovascular disorder characterized by recurrent attacks of headaches accompanied by symptoms such as nausea, vomiting, and photo- and phonophobia. Patients suffering from migraine with aura (MA) experience fully reversible neurological symptoms, usually before headaches within 60 min. Among them, visual aura is most common, and other sensory or speech disturbances are less frequent. Motor or brainstem functions are affected rarely. Two or more aura symptoms may occur, and they appear in succession [5]. This disorder highly depends on gender, since migraine is two-to-three times more prevalent in women than in men (21.0% of lifetime prevalence for women and 10.7% for men), especially during the reproductive age [6,7]. Migraine attacks commonly include visual symptoms, but the potential role of the retina in these symptoms is not well understood [8]. The retina, being part of the central nervous system (CNS) and containing a rich network of blood vessels, is uniquely positioned to provide insights into the vascular aspects of migraine. Research into the changes in retinal vessels observed in migraine patients has been growing, particularly with advances in imaging technologies such as optical coherence tomography angiography (OCTA). Migraine is believed to involve a combination of neural and vascular factors. The vascular component is particularly important for understanding the pathophysiology of migraine, and this is reflected by the retinal vasculature changes observed in migraineurs [9,10,11,12,13]. Retinal changes may be transient or permanent, and these vascular alterations may give clues to the pathophysiology of migraine attacks [8].

OCTA is an advanced imaging technique that allows for the non-invasive, high-resolution visualization of retinal blood vessels. This technology has been used to investigate the microvascular changes in the retina of migraine patients. OCTA can capture vascular density, blood flow, and capillary changes, providing a detailed picture of the retina’s vascular network [13,14]. This is particularly useful in assessing macular and peripapillary regions, which are critical areas for visual function. While retinal vascular changes detected via OCTA in migraine patients do not always lead to permanent visual impairment, they can possibly contribute to temporary visual disturbances such as visual auras and blurred vision during a migraine attack [7].

The aim of this study was to assess the vasculature and thickness of the macula using the OCTA technique in patients with migraine without aura (MO) and with aura (MA) compared to a healthy control group.

## 2. Material and Methods

### 2.1. Study Type

The study was conducted between July 2023 and November 2024 at the Chair and Department of General and Pediatric Ophthalmology of the Medical University of Lublin, Poland. The study followed the tenets of the Declaration of Helsinki and written approval of the Ethics Committee at the Medical University of Lublin, Poland (approval number KE-0254/207/10/2022). Written informed consent was obtained from each participant of this study.

### 2.2. Patient Characteristics

A total of 112 patients suspected of having MA or MO have been screened. A total of 71 patients were enrolled in the study; 41 patients were excluded from the analysis for reasons such as a non-confirmed migraine diagnosis, age under 18, or a history of ocular surgery and significant visual impairment. Among them, 20 individuals did not attend the ophthalmological examinations. Finally, the study included 37 right eyes of adult patients with MO and 34 of patients with MA. According to the International Classification of Disease version 10 (ICD-10), the patients were examined and recruited by two neurologists specializing in migraine management (KMS and AKSz). All screened participants underwent a clinical structured interview with a neurological examination and were diagnosed in accordance with the third edition of the International Classification of the Headache Disorders (ICHD-3) criteria [5]. Additionally, 34 healthy controls (HCs), who were willing to undergo OCT and OCTA scans of both eyes for our study, were recruited. HCs were recruited from outpatients attending a routine ophthalmological examination, students, and medical staff.

Inclusion criteria were as follows: participants aged 18–70 years with a diagnosis of MA, MO, or HCs with no history of migraine. The exclusion criteria for all subjects were as follows: (1) systemic diseases affecting the microvasculature, including diabetes mellitus and cardiovascular disorders, and neurodegenerative diseases; (2) alcohol and other psychoactive substance dependence; (3) any intra-ocular pathology (glaucoma, macular abnormalities, high ocular trauma, retinal detachment) or surgery; (4) refractive error greater than +3.00 or less than −6.00 diopters; (5) other diseases that may affect OCT or OCTA measurements; (6) poor cooperation and fixation during examination or failure to report for the ophthalmological examination; (7) no confirmation of the migraine diagnosis.

All patients underwent ophthalmological examinations: the best-corrected visual acuity (BCVA) in the Snellen decimal scale, slit-lamp biomicroscopy, intra-ocular pressure (IOP) measurement and pupillary reactions, objective refraction measurement, and fundus examination. Moreover, all patients underwent an OCT examination (Topcon, Tokyo, Japan), OCTA examination (Optovue, Fremont, CA, USA), and ultra-widefield fundus photography (Optos, Dunfermline, UK). All patients underwent visual field testing with a 24-2 SITA Fast (Humphrey Field Analyser II, Carl Zeiss Meditec Inc., Dublin, CA, USA). A detailed medical history was taken, including the BMI (body mass index) of the patient, symptoms perceived by patients, and other diseases.

### 2.3. OCTA Examination

OCTA examination was performed by the same ophthalmologist (P. L.). The OCTA scans were calculated using the integrated software algorithm, 70,000 A-scan/s. Identification and segmentation methods of areas were selected automatically. RPC density and RNFL thickness were measured via OCTA in the peripapillary area (4.5 × 4.5 mm). Moreover, macular thickness and vessel density (superficial and deep vascular complex) were measured with the same device on scans 6 × 6 mm for the macula. All sectors of the macula were analyzed (inferior, superior, nasal, temporal); furthermore, a comparison between superior and inferior hemifields of the macula was carried out in all the groups. Moreover, the foveal avascular zone (FAZ) was assessed in mm^2^. All scans were reviewed independently by two investigators (PL and KN) to ensure correct segmentation and sufficient imaging quality; a cut-off quality score of the OCTA scans was 7/10.

The following OCTA parameters were recorded: macula superficial vessel density (VD) whole image, macula superficial VD fovea, macula superficial VD parafovea (temporal, nasal, inferior, superior), macula deep vessel density (VD) whole image, macula deep VD fovea, macula deep VD parafovea (temporal, nasal, inferior, superior), thickness ILM-RPE macula whole image, thickness ILM-RPE macula fovea, thickness ILM-RPE macula parafovea (temporal, nasal, inferior, superior), FAZ, nonflow area, RNFL peripapillary thickness (temporal, nasal, inferior, superior), RPC VD capillary whole image.

### 2.4. Statistical Analysis

The database and statistical computations were carried out with STATISTICA 13.0 computer software (StatSoft, Kraków, Poland). The values of the analyzed measurable parameters were presented as the mean, median, and standard deviation (SD) and as counts and percentages in the case of non-measurable parameters. Both eyes of each patient were tested, but only the right eye was taken for analysis. This is in line with the suggestions made by Armstrong [15].

For the measurable variables, the normal distribution of the analyzed parameters was assessed using the Shapiro–Wilk test. The Kruskal–Wallis test using multiple comparison analysis was used to compare multiple independent groups. The Mann–Whitney test was used for the comparison of two independent groups (MA and MO). The level of significance of *p* < 0.05 indicated the existence of statistically significant differences.

## 3. Results

### 3.1. Patienst 

The study included 105 eyes of 105 individuals, of which 32.38% (*n* = 34) had MA, 35.24% (*n* = 37) had MO, and 32.38% (*n* = 34) were HCs. The MA group included 88.24% women (*n* = 30) and 11.76% men (*n* = 4), whereas in the MO group, 89.19% (*n* = 33) were women and 10.81% (*n* = 4) were men. The HCs consisted of HCs of 76.47% (*n* = 26) women and 23.53% (*n* = 8) men.

The migraine groups did not differ significantly in terms of age (Z = 0.72; *p* = 1.00). The differences were related to the comparison between the migraine groups and the control group (aura-control: Z = 3.41; *p* = 0.002; without aura-migraine: Z = 4.21; *p* = 0.0001) (Table 1).

In the MA group the body mass index (BMI) was significantly higher compared to the MO group. The observed differences were statistically significant (Z = −2,46; *p* = 0.01) (Table 2).

The most frequently reported aura symptoms in MA patients were as follows: scotomas (26.47%), partial vision loss (20.59%), facial/hand numbness (17.65%), spots (11.76%), and flashes (11.76%), while other symptoms (dizziness, speech difficulties, auditory disturbances, shadows, sensory disturbance, and double vision) were less common.

Patients in the MA group exhibited a higher prevalence of comorbid conditions compared with those in the MO group. The most common comorbidities in the MA group were Hashimoto’s thyroiditis (HT) (32.35%), hypertension (11.76%), and insulin resistance (11.76%). In the MO group, Hashimoto’s thyroiditis was the most frequent comorbidity (18.92%).

In the MA group, 50% had a family member with a history of migraine. In the MO group, 63.16% had a family member with a history of migraine.

The IOP value in the MA group was 15.14 ± 3.67 (Me = 15.00), while in the MO group, it was 15.39 ± 2.19 (Me = 15.00). Statistical analysis did not show significant differences between the groups in the IOP assessment (Z = −0.02; *p* = 0.98).

As a result of the statistical analysis, no significant differences were found in the assessment of visual field parameters between the migraine groups (*p* > 0.05) (Table 3).

### 3.2. OCTA Analysis

The conducted analysis did not reveal statistically significant differences in the assessment of individual RPC vessel density parameters (%) between the groups (*p* > 0.05), except for the evaluation of the inferior capillary sector, where values were significantly higher in the MO group compared to the HCs (Z = 3.10; *p* = 0.007) (Table 4).

The statistical analysis did not reveal any significant differences between women and men in the assessment of most OCTA disc RPC vessel density (%) parameters in both MA and MO groups, as well as in the HCs (*p* > 0.05). However, significant differences were found in the assessment of the RPC vessel density (%) in the temporal capillary (*p* = 0.04), with higher values observed in the men group, and in the superior capillary (*p* = 0.03), where higher values were found in the women group (Table 5).

Statistical analysis did not show a significant correlation between age and the individual RPC vessel density (%) parameters in the MA, MO, and HC groups (*p* > 0.05).

The statistical analysis did not show a significant relationship between BMI and individual RPC vessel density (%) parameters in the MA and MO groups (*p* > 0.05).

The statistical analysis revealed significant differences between the groups in the assessment of macular superficial vessel density (%) in the whole image (*p* = 0.04), with lower values observed in the MA group compared to both the HCs and the MO group (Table 6).

The studies demonstrated that the OCTA macular deep vessel density parameters were slightly lower in the MA and MO groups compared with the HCs. Statistical analysis did not reveal significant differences between the groups in the assessment of individual parameters (Table 7).

The statistical analysis did not show any significant differences in the assessment of macula OCT thickness ILM-RPE and FAZ parameters between the groups (*p* > 0.05).

Non-flow area values were comparable across all study groups. No statistically significant differences were observed between the groups (*p* = 0.27).

The statistical analysis did not reveal significant differences between women and men in the assessment of OCTA macula superficial vessel density (%) and OCT thickness ILM parameters, as well as FAZ, both in the MA and MO groups, as well as in the HCs (*p* > 0.05). The FAZ area was larger in both MA and MO groups compared to controls, although these differences were not statistically significant (*p* = 0.10).

The statistical analysis did not reveal a significant relationship between age and individual OCTA superficial macula vessel density (%) parameters, OCT thickness ILM, and FAZ in the MA, MO, and HC groups (*p* > 0.05).

The statistical analysis did not reveal any significant differences in RNFL parameter assessments between the migraine groups and the HCs (*p* > 0.05) (Table 8).

There was no statistical significance between age and individual RNFL parameters in the three study groups (*p* > 0.05), nor in the assessment of RNFL parameters between women and men within the migraine and HC groups (*p* > 0.05), nor between BMI and individual RNFL parameters in the MA and MO groups (*p* > 0.05).

The statistical analysis did not reveal a significant relationship between BMI and individual OCTA superficial macula vessel density (%) parameters and OCT thickness ILM in the MA and MO groups (*p* > 0.05).

However, significant differences were found in the MA group in the assessment of the FAZ parameter (R = −0.36, *p* = 0.04). As BMI increases, the FAZ value decreases. In the MO group, no relationship between FAZ and BMI was observed (*p* > 0.05) (Table 9).

## 4. Discussion

Since its introduction in 2015, OCTA has provided a growing volume of research, revealing detailed insights into the retinal microvascular architecture, providing details previously inaccessible through conventional imaging. Microvascular network alterations represent a key feature in the pathophysiology of many CNS disorders. 

In this study, OCTA demonstrated measurable alterations in both the macular and peripapillary retinal microvasculature in patients with migraine, with distinct patterns observed between MA and MO. The most notable finding was a significant reduction in the macular superficial vessel density (SVD) in the MA group compared to both MO patients and HCs. This observation indicates a more pronounced involvement of retinal microcirculation in MA and supports the concept of subtype-specific vascular differences. The reduced SVD observed in the MA group is consistent with current concepts of migraine pathophysiology, in which cortical spreading depression and transient cerebral hypoperfusion play a central role [16,17]. As the retinal and cerebral vasculature share an embryological origin and similar autoregulatory mechanisms, the retinal microcirculation may reflect a cerebral microvascular status [18,19,20]. Previous OCTA studies reported reduced macular vessel density in migraine patients, particularly in those with aura, although the magnitude and regional distribution of these changes have varied across studies or nonsignificant reductions [19,21,22,23,24,25,26]. The consistently greater impairment observed in MA compared with MO suggests that migraine with aura is associated with more substantial neurovascular dysregulation, potentially related to repeated episodes of hypoperfusion or endothelial dysfunction [27]. Our findings further reinforce this hypothesis by demonstrating significant differences between MA and MO subtypes.

However, other studies have reported different results. Chang et al. and Bingöl Kızıltunç et al. did not detect any significant differences among MA and MO patients and HCs in whole-image macular VD within the SCP, nor in any parameters of the DCP [25,28]. Güler et al. and Hamamci et al. reported no significant differences in VD measurements in the SCP and for the DCP between migraine patients and HCs [22,29], suggesting that an acute migraine attack does not affect retinal or peripapillary blood flow. Similarly, Ke et al., in their systematic review and meta-analysis, found no significant overall changes in macular VD in migraine patients compared with control subjects, highlighting the heterogeneity of OCTA findings across studies [30]. The lack of consistent, significant differences in retinal microvasculature between migraine and non-migraine groups can be attributed to several factors, including the transient nature of vascular changes and high autoregulation of the eyeball [25]. Retinal blood flow changes in migraine are often transient, occurring specifically during an attack due to vasospasm or cortical spreading depression, and may not leave permanent structural damage that is detectable between attacks. The eye is well-auto-regulated in terms of blood flow, which may prevent a migraine attack from affecting the retinal vascular density in a statistically significant way in a general population with migraine [31].

These discrepancies likely reflect methodological differences, including OCTA device specifications, segmentation algorithms, and population characteristics. Taken together, the literature indicates that the DCP is highly susceptible to subtle microvascular impairment in migraine, particularly in MA patients. Our findings align with this pattern, emphasizing that OCTA can detect early microvascular changes that precede structural retinal damage and may serve as a sensitive biomarker of subtype-specific vascular involvement in migraine.

Interestingly, MO patients demonstrated a significantly higher RPC vessel density in the inferior quadrant compared to HCs. This finding contrasts the reduced macular perfusion observed in MA and may reflect a compensatory or adaptive microvascular response in MO. Potential mechanisms include differences in autonomic nervous system regulation, neurovascular coupling, or local metabolic demand between migraine subtypes, which could lead to regional variations in retinal perfusion [32,33].

Previous OCTA investigations have reported heterogeneous changes in peripapillary microvascular density among migraine patients, supporting the notion that microvascular alterations are not uniform across retinal regions or migraine phenotypes. For example, Chang et al. described reduced peripapillary VD predominantly in MA, with no significant changes in MO [25]. The same observations were reported by Karahan et al. in their study [34]. Liu et al., in a systematic review and meta-analysis, reported that perfusion density in the RPC segment was decreased inside the optic disc in migraine cohorts, but overall peripapillary findings varied across studies, emphasizing regional variability in retinal perfusion abnormalities [9]. Conversely, Güler et al. did not find significant differences in flow areas of the optic nerve head, vitreous, peripapillary capillary network, or choroidal segments between migraine patients and HCs. This data indicates preserved perfusion in these regions, suggesting that the sample size, disease duration, and attack frequency may influence detectable vascular alterations [29]. These equivocal results highlight the complexity of retinal microvascular responses in migraine and underscore the importance of considering both the migraine subtype and retinal location when interpreting OCTA measurements.

Additionally, our research compared RPC vessel density in relation to women and men groups. Gender-related differences in retinal microvascular parameters have been reported in healthy subjects, with OCTA demonstrating age and sex effects on RPC vessel density [35,36]. Su et al. found significant sex-related variations in the retinal capillary density using OCTA, suggesting physiological differences that should be considered when interpreting microvascular metrics [35]. Moreover, gender differences in ocular blood flow and vascular regulation have been attributed in part to hormonal influences, with estrogen potentially modulating vasodilation and perfusion [37]. Additional studies using laser speckle flowgraphy support the presence of sex differences in optic nerve head blood flow parameters [38]. In line with these observations, our finding of a higher temporal RPC vessel density in men and higher superior RPC vessel density in women may reflect intrinsic sex-related vascular variations, reinforcing the need to account for sex as a covariate in retinal perfusion studies. The higher prevalence of migraine in women and its hormonal modulation further support the relevance of sex-specific microvascular regulation.

Despite these vascular alterations, no significant differences were found in the RNFL thickness, FAZ area, or non-flow area between the studied groups. These findings suggest that migraine-related microvascular changes may occur in the absence of overt structural retinal damage, particularly in younger patients or in early stages of the disease. In this area, it would be worthwhile to perform long-term observations to assess changes over time. Previous studies have similarly reported preserved RNFL thickness in migraine patients, supporting the hypothesis that vascular dysregulation precedes neurodegenerative changes [39,40]. However, Feng et al. and Ke et al., in their meta-analysis, found that RNFL thickness in the migraine patients was thinner than that in the HC group [30,41]. Ascaso et al. summarized OCT findings in chronic migraine and reported that RNFL thinning may occur in a subset of migraine patients, particularly in chronic migraine and MA, suggesting possible subclinical optic nerve axonal damage related to recurrent ischemia. However, the authors emphasized that RNFL findings across studies are inconsistent, with some showing global thinning and others only sectoral changes [42]. Chang et al. reported that the MA patients demonstrated borderline thinner average RNFL when compared with the HCs. In addition, the superior RNFL was marginally thinner in the MA participants when compared with the HCs. There was no significant difference among the groups in the thickness of other quadrants of the RNFL [25]. Liu et al. found that the thickness of the RNFL of patients with MA and MO decreased significantly in most regions [9]. Regarding the size of the FAZ, no significant differences were observed between migraine groups (both MA and MO) and HCs. Similar findings have been reported by other authors [24,28,34]. However, Hamamci et al. [22], Chang et al. [25], Shah et al. [21], Liu et al. [9] Taşli et al. [26], and Hamurcu et al. [14] reported opposite results, demonstrating that FAZ was significantly larger in the MA group compared with HCs.

A significant inverse correlation between the BMI and FAZ area was observed in the MA group, indicating that a higher BMI may exacerbate retinal microvascular alterations in patients with MA. Although this finding was statistically significant, the correlation coefficient indicates that the relationship is very low. This suggests that retinal blood vessel changes found in migraineurs are likely caused by the underlying pathophysiology of the migraine itself, rather than by the patient’s weight or body composition. Previous studies reported obesity-related changes in retinal and choroidal microvasculature, including reduced deep FAZ in individuals with a higher BMI [43], which suggested that increased body mass may have some effects on the choroidal and retinal microvascular structure. Consistently, BMI was significantly higher in the MA group compared to the MO group, suggesting a potential interaction between metabolic status and migraine-related vascular changes.

In the present study, no statistically significant differences were observed in visual field parameters between migraine patients and healthy controls. This finding is in line with reports suggesting that functional visual field alterations are not consistently present in migraine, particularly in patients without a coexisting optic nerve pathology. Although earlier studies, such as that by Comoğlu et al., described glaucomatous-like visual field defects in a subset of migraine patients, those alterations were mainly observed in individuals with long-standing disease or additional risk factors for optic nerve damage [44]. The absence of visual field abnormalities in our cohort may therefore indicate preserved functional integrity of the visual pathway, despite detectable microvascular changes on OCTA. This supports the concept that retinal microcirculatory alterations in migraine may precede functional deficits and remain subclinical, at least in patients without advanced or chronic disease. Similar findings were reported in studies by Cankaya et al., Demircan et al., and Salman et al. [45,46,47].

Based on the medical history obtained from patients, the most common comorbidities in the MA group were Hashimoto’s thyroiditis (HT) (32.35%), hypertension (11.76%), and insulin resistance (11.76%). In the MO group, HT was the most frequent comorbidity. The high prevalence of Hashimoto’s disease among migraine patients may suggest a potential autoimmune component or shared pathophysiological mechanisms, such as endothelial dysfunction or chronic low-grade inflammation, contributing to both conditions. Similarly, Yin et al. discovered that thyroid diseases were more prevalent in the migraine group than in the HCs. (7.2% vs. 2.8%) [48]. Nowaczewska et al. found that HT is highly prevalent among individuals with migraine. Out of 928 patients included in their study, 11.4% were diagnosed HT [49]. The autoimmune nature of HT may help explain its association with migraine. Prior research has demonstrated that headaches, including migraine, occur more frequently in systemic autoimmune disorders, where a shared underlying factor such as endothelial dysfunction appears to play a central role [50]. Also, MA carries a higher burden of comorbidities in comparison to MO.

Based on medical histories, 50% of patients in the MA group and 63.16% in the MO group reported a family history of migraine, supporting the well-established familial and genetic predisposition to migraine. Family and twin studies have demonstrated that migraine has a substantial heritable component, with common forms such as MA and MO exhibiting polygenic risk and locus-specific associations that contribute to susceptibility [51,52].

The limitation of our study is that the mean age the control group is lower than the group of patients with migraine. The main reason was recruitment challenges, as younger patients were more accessible and willing to participate in the study. Nevertheless, the statistical analysis showed no correlation between age and OCTA parameters in this study. The prevalence of migraine is consistently higher across all ages, typically peaking during the reproductive years. Another shortcoming of this study is that we did not take into account the axial length and refraction of patients into the statistical analysis. It is already known that increased axial length and higher myopia strongly correlate with reduced OCTA-measured vessel density, particularly in the macula and superficial retinal layers [53]. As the eye elongates, the retina stretches, causing a decrease in the vascular network density. However, the exclusion criterion in this study was refractive error greater than +3.00 or less than −6.00 diopters, so highly myopic patients have not been included in the study.

However, the distinct microvascular alterations observed between MA and MO in our OCTA analysis suggest that genetic predisposition alone may not fully account for the vascular phenotype. Taken together, these observations highlight a complex interplay between familial/genetic risk and microvascular involvement in migraine, where genetic predisposition may set the stage for disease expression, but vascular and systemic modifiers influence phenotypic severity and subtype-specific retinal changes. Further studies with larger cohorts are warranted.

## 5. Conclusions

In summary, OCTA revealed subtype-specific retinal and peripapillary microvascular alterations in migraine, with MA showing reduced SVD and DCP, while MO exhibited increased RPC in the inferior quadrant. Sex, BMI, and comorbidities influenced microvascular patterns, and no structural retinal changes were detected. These findings suggest that OCTA can detect early and subtle microvascular changes in migraine, particularly in MA, before structural retinal damage occurs, potentially serving as a noninvasive biomarker for subtype-specific vascular dysregulation.

## Figures and Tables

**Table 1:** Age assessment in the study groups.

Group	Mean	Std. Dev.	Lower Quartile	Median	Upper Quartile
Migraine with aura	37.47	14.11	24.00	38.00	45.00
Migraine without aura	38.95	12.32	27.00	40.00	49.00
Healthy controls	27.00 *	8.69	22.00	23.00	27.00

Statistical analysis: H = 19.95; *p* < 0.0001 *.

**Table 2:** BMI assessment in the migraine groups.

Group	Mean	Std. Dev.	Lower Quartile	Median	Upper Quartile
Migraine with aura	25.02 *	4.05	22.72	24.54	27.22
Migraine without aura	22.82	3.74	20.43	21.84	24.77

Statistical analysis: Z = −2.46; *p* = 0.01 *.

**Table 3:** Assessment of the visual field parameters.

Visual Field Parameters	Migraine with Aura			Migraine Without Aura			Statistical Analysis	
	Mean	Median	Standard Deviation	Mean	Median	Standard Deviation	Z	*p*
Visual field index	99.00	99.50	1.41	98.68	99.00	1.38	−1.06	0.29
Mean deviation	−0.40	−0.51	1.43	−0.82	−0.37	1.55	−0.63	0.53
Pattern standard deviation	1.63	1.49	0.76	1.58	1.45	0.38	0.55	0.58

**Table 4:** Assessment of radial peripapillary capillary (RPC) vessel density parameters (%) in the study groups.

RPC Vessel Density (%) Capillary	Migraine with Aura			Migraine Without Aura			Healthy Controls			Statistical Analysis
	Mean	Median	Standard Deviation	Mean	Median	Standard Deviation	Mean	Median	Standard Deviation	
Whole image	50.87	50.75	2.74	51.58	51.70	2.22	50.86	50.65	1.80	H = 3.19. *p* = 0.20
Superior	53.38	55.00	4.95	53.95	54.00	3.88	53.00	53.00	3.02	H = 2.45. *p* = 0.29
Inferior	55.59	56.00	4.45	56.78	56.00	3.04	54.32	55.00	2.98	H = 9.87. *p* = 0.007 *
Nasal	48.94	50.50	5.14	50.32	50.00	3.50	49.59	49.50	3.12	H = 1.24. *p* = 0.54
Temporal	56.21	56.00	2.82	55.84	56.00	3.45	54.76	55.00	2.98	H = 4.23. *p* = 0.12

Statistical analysis: Z = 3.10; *p* = 0.007 *.

**Table 5:** Assessment of the radial peripapillary capillary (RPC) vessel density % parameter across groups in regard to patient gender.

OCT-A Parameter	Females			Males			Statistical Analysis	
	Mean	Median	Standard Deviation	Mean	Median	Standard Deviation	Z	*p*
Migraine with aura								
RPC vessel density (%) capillary whole image	50.83	50.95	2.86	51.18	50.60	1.73	−0.13	0.89
RPC vessel density (%) capillary SUPERIOR	53.20	54.50	4.98	54.75	57.00	5.25	−0.94	0.35
RPC vessel density (%) capillary INFERIOR	55.50	56.00	4.70	56.25	56.50	1.71	−0.32	0.75
RPC vessel density (%) capillary NASAL	48.90	50.50	5.31	49.25	49.50	4.27	0.03	0.98
RPC vessel density (%) capillary TEMPORAL	55.87	55.50	2.78	58.75	58.50	1.71	−2.03	0.04
Migraine without aura								
RPC vessel density (%) capillary whole image	51.56	51.60	2.27	51.78	51.95	2.06	−0.37	0.71
RPC vessel density (%) capillary SUPERIOR	54.33	55.00	3.92	50.75	51.00	1.26	2.18	0.03
RPC vessel density (%) capillary INFERIOR	56.85	56.00	3.05	56.25	56.50	3.30	0.12	0.90
RPC vessel density (%) capillary NASAL	50.30	50.00	3.60	50.50	50.50	2.89	−0.10	0.92
RPC vessel density (%) capillary TEMPORAL	55.64	55.00	3.56	57.50	57.00	1.91	−1.22	0.22
Control group								
RPC vessel density (%) capillary whole image	51.12	50.75	1.49	50.00	49.65	2.52	1.58	0.11
RPC vessel density (%) capillary SUPERIOR	53.27	53.00	2.78	52.13	53.00	3.76	0.51	0.61
RPC vessel density (%) capillary INFERIOR	54.46	54.50	2.50	53.88	55.00	4.39	−0.14	0.89
RPC vessel density (%) capillary NASAL	49.96	50.00	2.86	48.38	48.50	3.81	1.01	0.31
RPC vessel density (%) capillary TEMPORAL	54.50	54.50	2.53	55.63	56.50	4.21	−1.18	0.24

**Table 6:** Assessment of OCTA macular superficial vessel density (%) in the parafoveal region (%) across groups.

OCTA Macular Superficial Vessel Density (%)	Migraine with Aura			Migraine Without Aura			Healthy Controls			Statistical Analysis
	Mean	Median	Standard Deviation	Mean	Median	Standard Deviation	Mean	Median	Standard Deviation	
Whole image	50.39	51.15	3.18	51.98	52.60	3.54	51.86	52.30	2.87	H = 6.21. *p* = 0.04
Fovea	21.85	21.95	6.44	20.96	21.20	7.23	23.35	22.95	5.44	H = 2.42. *p* = 0.30
Temporal	52.31	53.35	4.00	53.97	54.90	3.02	53.64	54.25	3.56	H = 3.09. *p* = 0.21
Superior	54.41	55.10	4.72	55.68	56.80	4.26	55.67	56.00	3.59	H = 2.46. *p* = 0.29
Nasal	52.30	53.40	3.89	53.84	54.70	3.79	53.83	53.70	3.27	H = 3.81. *p* = 0.15
Inferior	53.10	53.80	5.19	54.58	55.60	4.61	54.31	54.95	4.57	H = 1.94. *p* = 0.39

**Table 7:** Evaluation of OCTA macular deep vessel density parameters (%) across groups.

OCTA Macular Deep Vessel Density (%)	Migraine with Aura			Migraine Without Aura			Healthy Controls			Statistical Analysis
	Mean	Median	Standard Deviation	Mean	Median	Standard Deviation	Mean	Median	Standard Deviation	
Whole image	52.37	53.25	5.46	54.47	55.90	5.37	54.83	56.40	5.38	H = 4.60, *p* = 0.10
Fovea	40.52	40.20	6.14	37.82	38.50	7.62	41.59	41.85	5.22	H = 5.69. *p* = 0.06
Parafovea temporal	58.26	58.85	3.67	59.91	60.00	2.76	59.04	59.75	4.26	H = 3.46. *p* = 0.18
Parafovea superior	56.66	57.60	4.66	58.30	58.80	3.92	58.16	59.55	4.00	H = 2.92. *p* = 0.23
Parafovea nasal	57.68	57.75	3.87	59.15	59.10	2.65	59.03	59.55	3.97	H = 2.57. *p* = 0.28
Parafovea inferior	55.39	55.55	5.17	57.63	58.30	3.72	57.03	58.10	4.87	H = 3.64. *p* = 0.16

**Table 8:** Assessment of the retinal nerve fiber layer (RNFL) parameters (µm) in the study groups.

RNFL Thickness (um)	Migraine with Aura			Migraine Without Aura			Healthy Controls			Statistical Analysis
	Mean	Median	Standard Deviation	Mean	Median	Standard Deviation	Mean	Median	Standard Deviation	
Peripapillary	111.85	114.50	12.95	112.81	111.00	11.35	115.12	113.00	12.79	H = 1.14, *p* = 0.57
Superior	131.62	131.00	16.85	130.65	128.00	17.83	136.12	135.50	17.44	H = 1.55, *p* = 0.46
Inferior	143.79	147.00	20.46	143.30	144.00	16.29	145.26	146.00	17.11	H = 0.28, *p* = 0.87
Nasal	100.50	101.50	18.93	103.35	101.00	15.66	106.50	102.50	21.13	H = 0.95. *p* = 0.62
Temporal	75.21	75.00	9.19	75.70	73.00	12.92	75.00	73.00	9.60	H = 0.18, *p* = 0.91

**Table 9:** Assessment of the association between BMI and the parameters macula superficial vessel density (%) and OCT thickness ILM in the groups with migraine with aura and without aura.

Parameters	Statistical Analysis	
	R	*p*
Migraine with aura		
Macula superficial vessel density (%) whole image	0.00	1.00
Macula superficial vessel density (%) fovea	0.31	0.09
OCT thickness ILM-RPE macula (um) whole image	0.13	0.47
OCT thickness ILM-RPE macula (um) fovea	0.30	0,09
FAZ mm^2^	−0.36	0.04
Migraine without aura		
Macula superficial vessel density (%) whole image	−0.11	0.54
Macula superficial vessel density (%) fovea	−0.06	0.76
OCT thickness ILM-RPE macula (um) whole image	0.05	0.80
OCT thickness ILM-RPE macula (um) fovea	0.15	0.42
FAZ mm^2^	−0.01	0.95

## Data Availability

The original contributions presented in this study are included in the article. Further inquiries can be directed to the corresponding author.

## Abbreviations

The following abbreviations are used in this manuscript:

MA	migraine with aura
MO	migraine without aura
HCs	healthy controls
FAZ	foveal avascular zone
ILM	inner limiting membrane
OCT	optical coherence tomography
OCTA	OCT angiography
HT	Hashimoto Thyroiditis
SVD	superficial macular vessel density
DCP	deep capillary plexus
VD	vessel density
RPC	radial peripapillary capillary
CNS	central nervous system
